# Next of kin’s quality of life before and after implementation of a knowledge-based palliative care intervention in nursing homes

**DOI:** 10.1007/s11136-019-02268-9

**Published:** 2019-08-14

**Authors:** Christina Bökberg, Lina Behm, Gerd Ahlström

**Affiliations:** Department of Health Sciences, Faculty of Medicine, Lund University, P.O. Box 157, 221 00 Lund, Sweden

**Keywords:** End-of life, Family member, Long-term care, Older persons, Significant others, Staff education

## Abstract

**Purpose:**

The purpose of this study was to evaluate whether an educational palliative care intervention improved the quality of life for next of kin to older persons in nursing homes.

**Methods:**

Altogether, 90 next of kin in the intervention group and 105 next of kin in the control group were included. Data were collected using the WHOQOL-BREF questionnaire, answered before and 3 months after the intervention was completed. Descriptive and comparative analyses were performed.

**Results:**

This study found a statistically significant increase in the Physical health subscale in the intervention group but not in the control group. In contrast, the General health score decreased in the control group but not in the implementation group. Furthermore, we found an increase in the item *able to perform activities of daily living* in the intervention group and a decrease in the item *energy and fatigue* in the control group.

**Conclusion:**

The results indicated small statistical changes regarding next of kins’ QoL in favour of the intervention. Lessons learned from the study for future research are to include next of kin as participants at meetings about next of kin and to include more meetings about the theme next of kin. Both approaches would bring a stronger focus on the family-centred care aspect of the intervention into the education component, which this study indicates the need for.

**Trial registry:**

Trial registration NCT02708498. Date of registration 26 February 2016.

## Introduction

The palliative care approach emphasises person- and family-centred care and focuses on the person and his/her family, not just the disease [1]. A main outcome for palliative care is to promote quality of life (QoL), not merely for those persons suffering from life-threatening conditions but also for their next of kin [2]. To promote QoL at the end-of-life, palliative care has a holistic approach consisting of physical, psychosocial and spiritual dimensions [1]. Palliative care should include all persons with life-threatening illnesses, irrespective of age, diagnosis and context of care. Although it is the frailest and oldest persons who live in nursing homes, older persons in nursing homes and their next of kin have received far less palliative care than younger people [1–3].

The next of kin have unique knowledge of the older person, and it is therefore important for staff to actively communicate with next of kin and incorporate them into the care for the proper planning of the person’s remaining life [2, 4]. There is a prerequisite for staff to be able to adapt information and support for the family based on changing needs during the older persons’ disease trajectory [2]. Wallerstedt et al. [5] found that next of kin expect staffs to recognise not only the next of kins’ importance for the older person’s well-being but also their needs for information and guidance. However, previous results have found that end-of-life discussions are rarely performed with the next of kin [6] and that next of kin are initially included in the care in the very late phase [7]. When the palliative approach is lacking, the next of kin are excluded from information and decision making concerning their relatives’ care, which could increase frustration, anxiety and worrying [5] and thereby have a negative impact of their QoL.

Next of kin to an older person in a nursing home can experience conflicting or mixed feelings, such as guilt, relief and remorse, between their own needs and their sense of responsibility for the older person’s well-being and care [8, 9]. Another dilemma could be balancing between taking part in the care, while at the same time, leaving the responsibility of the care to the staff [5]. Such feelings and distress could decrease the QoL in next of kin, and these feelings and distress need to be considered and reduced by the staff.

For staff to be able to support and promote QoL in the next of kin to older persons in nursing homes, the staff need to adapt palliative care efforts to the specific and complex needs of the families [1]. However, it seems that staff in nursing homes do not have sufficient knowledge, skills and training in managing symptoms and other aspects of palliative care [10, 11] and that staff in nursing homes have requested more education and knowledge in palliative care [12, 13] as well as training in how to meet the emotional and existential needs of the next of kin [7]. Therefore, the KUPA project (an acronym for the Swedish title: KUnskapsbaserad PAlliativ vård, which in English is “Knowledge-based Palliative Care”] implemented an educational palliative care intervention directed to staff and managers in 20 nursing homes in Sweden. One of the main outcomes was to evaluate whether the intervention had any influence on QoL in next of kin for older persons in nursing homes [14].

## Purpose

The purpose of this study was to evaluate whether an educational palliative care intervention improved the quality of life of next of kin to older persons in nursing homes.

## Methods

### Design

During 2015–2017, the KUPA project was conducted as a complex intervention with a non-randomised crossover design. In 20 nursing homes, in two different counties in southern Sweden (County A and County B), the knowledge-based palliative care intervention was implemented. First, the intervention was implemented in ten nursing homes in County A, while ten nursing homes in County B served as a control group. Second, the ten control nursing homes in County B implemented the intervention, and ten new nursing homes in County A, which had not received the intervention, were chosen as a control group. The intervention of this crossover experimental design was then evaluated before and after the intervention [14]. This study is a part of that evaluation.

### Setting

In Sweden, care and services for older persons are primarily a public-financed and publicly provided responsibility. The ‘ageing in place’ ideology implies that older persons remain living in their own homes despite severe illnesses [15]. In addition to service staff working in older people’s homes, there are many people who take care of their older relatives even if there is no formal responsibility. To support ‘ageing in place’, Swedish municipalities have been obliged by law since 2009 [16] to offer and facilitate support for those who care for a close relative who is older, has a long-term illness or has long-standing disabilities. The frailest older people live in nursing homes, and they have their own lease contracts, which means that nursing homes are also formally ‘a home’. Seventy-one percent of all people who died in Sweden in 2017 were 75 years or older [17], and 38% of all deaths in Sweden occurred in nursing homes [18]; these findings suggest that it is staff in the nursing homes who largely provide end-of-life care to frail older persons.

### The knowledge-based palliative care intervention

#### Feasibility/pilot intervention study

A feasibility/pilot intervention study was undertaken before the educational intervention started. This study consists of six 2–3-h seminars at four nursing homes using the educational booklet as study material for the participants. The feasibility of the pilot intervention resulted in a final version of the intervention, which consisted of a series of five 2-h seminars about palliative care for staff and managers.

#### The final intervention

The final intervention consisted of a series of seminars based on a booklet designed within the KUPA project consisting of five themes: (1) the palliative approach and dignified care, (2) next of kin, (3) existence and dying, (4) symptom relief and (5) collaborative care [19]. The booklet was based upon scientific articles and two knowledge documents [20, 21] and was developed in collaboration with staff, informal caregivers and patients from different care settings. The seminars addressed the knowledge and skills that have been deemed necessary and should be translated into routine practice. Each seminar group was led by two registered nurses, and each group consisted of a mix of 8–10 staff and managers. The staff and managers were recruited consecutively to the intervention in equal numbers from both the intervention nursing homes and the control nursing homes. The staff had seminars every 4–5 weeks for 6 months, and each seminar lasted approximately 2 h [14].

### Sample

**Fig. 1:**
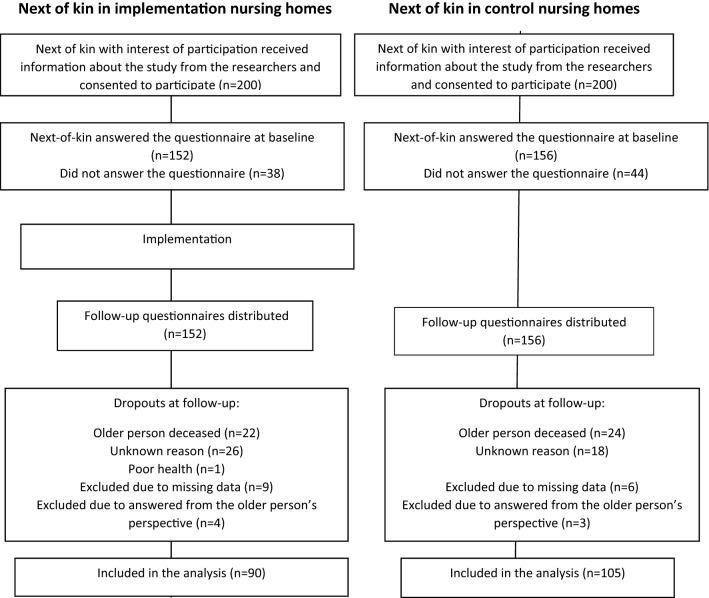
Flow chart showing the inclusion procedure for the study participants

The selection of nursing homes was made through voluntary participation and resulted in a mixture of both larger (> 100 resident) and smaller (< 25 residents) nursing homes, from both urban and rural areas. The inclusion criteria were a next of kin who had a relation to an older person living in one of the participating nursing homes but was not necessarily a family member or a relative [14]. The next of kin were recruited consecutively in equal numbers from both the intervention nursing homes and the control nursing homes. One designated contact person at each of the included nursing homes informed the next of kin, if he or she had fulfilled the inclusion criteria, of the KUPA project and asked about interest for participation in the study. In total, 400 next of kin consented to participate, whereas 308 completed the questionnaires at baseline. The dropouts consisted of 113 next of kin (Fig. 1).

Altogether, 90 next of kin in the intervention group and 105 in the control group participated in this study before and after the intervention. No statistically significant differences were detected between the two groups at baseline. Background characteristics are provided in Table 1.

**Table 1:** Characteristics of the next of kin in the intervention and the control groups at baseline

Background variable	Intervention group (*n* = 90)	Control group (*n* = 105)
Age		
Years, median (range)	65 (29–92)	65 (45–96)
Gender, *n* (%)		
Female	66 (73)	84 (80)
Civil status, *n* (%)		
Married	71 (79)	86 (78)
Unmarried	17 (19)	17 (16)
Widow/widower	2 (2)	2 (6)
Relation to older person, *n* (%)		
Wife/husband	17 (19)	20 (18)
Son/daughter	66 (73)	73 (66)
Son- or daughter-in-law	1 (1)	2 (2)
Sibling	2 (2)	4 (4)
Other	3 (4)	4 (4)
Missing	1 (1)	2 (2)
Frequency of visits, *n* (%)		
Every day	7 (8)	10 (9)
≥ 1 per week	65 (72)	69 (63)
≥ 1 per month	5 (17)	23 (21)
Sometimes per year	1 (1)	0 (0)
Missing	2 (2)	3 (7)

### Data collection

If the next of kin were willing to participate, the contact person handed over the contact information to the research team. Three researchers were responsible for contacting the participants via telephone where they provided further information about the project and this particular study (Fig. 1). Those who agreed to participate received by mail written information about the study, written informed consent form, coded questionnaires and stamped and addressed envelopes for reply by mail.

At the time of the follow-up assessment, but before sending the same questionnaire by mail, the research team contacted the contact person at the nursing homes to obtain information regarding any changes that had occurred over the relevant time span. When the questionnaires were returned, the project administrator registered the coded questionnaires and written informed consent separately.

### Measurement

The data collection was based on the abbreviated version of the WHOQOL-100, the World Health Organization Quality of Life (WHOQOL-BREF) questionnaire, answered before (baseline) and 3 months after (follow-up) the intervention was completed, i.e. 9 months after baseline. The same time intervals were used for the control nursing homes. The WHOQOL-BREF consists of 26 questions that assessed individual facets related to QoL. Of these 26 questions, there was one question related to Overall quality of life and one question related to General health. The remaining questions of the instrument encompassed four domains: (1) *Physical health*, containing seven items; (2) *Psychological,* containing six items; (3) *Social relationships,* containing three items and (4) *Environment,* containing eight items. Informants were asked to respond on a five-point positive-directed Likert scale. The total possible score of the scale ranged from 26 to 130, where higher values indicated a higher quality of life [22]. The psychometric properties of the WHOQOL-BREF items in next of kin to older persons living in nursing homes have been demonstrated to have acceptable reliability and validity (Rosén, Ahlström, Lexen, unpublished). Due to the Regional Ethics Review Board in Lund (reference number: 2015/4), the item ‘Sexual activity’ in the domain social relationships needed to be removed. The WHO has given their permission to exclude the item to GA (PI in this project). Thus, the total possible score in this study ranged from 25 to 125.

### Statistical analyses

The data were analysed using within-group comparisons (i.e. the follow-up data were compared to the baseline data in each of the intervention and control groups). The selection of statistical methods was based on whether the data, not including the descriptive statistics, were distributed normally and the scale level of the instruments. The Mann–Whitney U-test was applied to compare the baseline characteristics of the two groups. A Wilcoxon signed-rank test was used to make within-group comparisons. Either the Pearson Chi-square test or Fisher’s exact test was performed if any expected cell value was less than 5. Analyses were performed using IBM SPSS Statistics version 24. A two-tailed *p* value of < 0.05 was regarded as statistically significant. Missing data on single items were replaced by the mean score for that item in the group [22].

### Ethical considerations

This study was guided by the ethical principles for medical research, the Declaration of Helsinki [23]. To generate valuable knowledge while minimising harmful consequences, several actions were taken. The participants’ autonomy was respected as they received oral and written information about the aim and design of the study, voluntary participation and the right to withdraw from the study at any time without suffering from any consequences before signing the informed consent. The participants were guaranteed confidentiality since their personal data are kept in locked closets and retained only by the PI (GA) of the KUPA project. Confidentiality was further increased by encoding the questionnaire data, making it not possible to identify individuals and by presenting the findings at the group level. The Regional Ethics Review Board in Lund approved the KUPA project (reference number: 2015/4), and the project is registered as Trial registration: NCT02708498.

## Results

There was a statistically significant increase in the mean score (+ 0.26) on the subscale *Physical health* in the implementation group, but not in the control group, when the data after the intervention period were compared with the baseline data within the respective groups. In contrast, *General health* declined (mean score changes of − 0.15) in the control group but not in the implementation group (Table 2).

**Table 2:** Intervention group (*n *= 90) and control group (*n *= 105) analyses of WHOQOL-BREF before and after implementation

Domains and items	Intervention group Baseline Median (Q1–Q3)	Intervention group Follow-up Median (Q1–Q3)	*p* value^b^	Control group Baseline Median (Q1–Q3)	Control group Follow-up Median (Q1–Q3)	*p* value^b^
**Total score** (25–*125*)^a^	95 (90–102)	94 (87–102)	0.346	105 (96–111)	103 (96–111)	0.307
**General QoL** (1–*5*)^a^	4 (4–5)	4 (4–5)	1.000	4 (4–5)	4 (4–5)	0.113
**General health** (1–*5*)^a^	4 (3–4)	4 (3–4)	0.558	4 (4–5)	4 (3–5)	**0.024**
**Physical health** (7–35)^a^	16 (14–18)	16 (15–18)	**0.033**	17 (15–18)	16 (14–18)	0.289
Abilities to perform activities of daily living	4 (3–5)	4 (4–5)	**0.027**	4 (3–5)	4 (3–5)	0.560
Dependence on medicinal substances and medical aids						
Energy and fatigue	4 (3–4)	4 (3–5)	0.177	4 (3–5)	4 (3–4)	**0.045**
Mobility						
Pain and discomfort						
Sleep and rest						
Work Capacity						
**Psychological** (6–30)^a^	16 (14–17)	16 (14–17)	0.887	16 (15–17)	17 (15–18)	0.969
Bodily image and appearance						
Negative feelings						
Positive feelings						
Self-esteem						
Spirituality/religion/personal beliefs						
Thinking, learning memory and concentration						
**Social relationships** (2–10)^a^	4 (4–5)	4 (4–5)	0.170	4 (4–5)	4 (4–5)	0.530
Personal relationships						
Social support						
**Environment** (8–40)^a^	17 (15–18)	16 (15–18)	0.368	17 (15–18)	17 (15–18)	0.862
Financial resources						
Freedom, physical safety and security						
Health and social care: accessibility and quality						
Home environment						
Information						
Participation and opportunities for recreation/leisure activities						
Physical environment pollution/noise/traffic/climate)						
Transport						

Significant values are given in bold

*Q1* first quartile, *Q3* third quartile

^a^Italicised score is the most favourable score

^b^Wilcoxon Signed-Rank Test

In addition, statistically significant changes were found at the item level. First, in the intervention group, an increase (mean score changes of + 0.25) in the item *able to perform activities of daily living* in the *Physical health* subscale was found. Second, in the control group, the score of the item *energy and fatigue* decreased (mean score changes of − 0.13) in the *Physical health* subscale. This decrease was not found in the intervention group (Table 2).

## Discussion

This study found very small statistical changes regarding next of kins’ QoL on two subscales related to health after an attempt to implement a palliative care intervention in nursing home settings in Sweden. In addition, at the item level, a small significant increase in the item *able to perform activities of daily living* was obtained in the intervention group, and a decrease in *general health* as well as on the item *energy and fatigue* was obtained in the control group. Pessimism towards negative results or non-significant results is seldom discussed among researchers, and negative results remain a low priority for publication, as the current scientific culture clearly favours positive results. However, including and reporting non-significant and/or negative results may improve scientific thinking, improve future studies and be necessary for a more complete scientific understanding [24].

This study is one of few published evaluations that presents the effects of implementing palliative care in a nursing home setting. A review of the literature [25] found only three studies, and only one study reported outcomes regarding the next of kin. That study [26], performed in the USA, concluded that implementation of palliative care had the potential to increase next of kins’ perceptions of the quality of care. This is in line with our assumption when designing this study, which was that it is reasonable to believe that the implementation of a palliative care approach, emphasising person- and family-centred care, would affect not only the staff and the resident but also the next of kin. Palliative care aims at involving and supporting the next of kin in the care process [1], and the fact that next of kin of residents at nursing homes want to be involved in the end-of-life care at the nursing home had been shown in several studies [27–29]. However, conversations and guidance from the staff are often lacking [28, 29]. The modest result of this study thus provokes questions about the effectiveness of the implementation as well as methodological issues, which is discussed below.

The effect of the implementation is dependent on several factors, such as (1) the effectiveness of the implementation strategies, (2) the characteristics of the implementation object, (3) the characteristics of the implementers, (4) the target population and (5) the context of the implementation [30, 31]. The effectiveness of the implementation strategy (factor 1) in terms of an educational intervention with only one meeting about next of kin is in need of consideration. That meeting aimed to increase knowledge and understanding of the situation and role of the next of kin and to consider how their need for support can be better met. The task after the meeting was to talk to one next of kin about their preferences for involvement and support. This meeting that lasted for 1.5 h, along with the knowledge that a palliative care approach in itself means family-centred care [1], was evidently not enough. One proposal for the future is to include next of kin in the intervention group as participants at the meeting about next of kin. Another suggestion is to include more meetings about the theme next of kin. Both approaches would bring a stronger focus on the family-centred care aspect of the intervention into the education component, which this study indicates the need for.

The characteristics of the implementation object (factor 2) are important, and in this project the implementation object is the evidence-based framework of palliative care described in two national documents produced by the National Board of Health and Welfare [21] and the Regional Co-operative Cancer Centre [20]. An ethnographic study halfway into the KUPA project indicated that the knowledge-based palliative care was perceived as new knowledge by the nurse assistants [32], who have a short, often half a year, pre-university education. They appreciated the knowledge and tools they received, but at the same time, this finding meant that there were barriers, such as limited time and emotional stress, to having conversations about death. The complexities experienced by nurse assistants may have negatively influenced the degree of implementation of the object, i.e. palliative care.

The effect of the implementation is also dependent on the characteristics of the implementers (factor 3), which in this study were the staff and the managers of the organisations. According to the Organizational Readiness to Chance theory (ORC) [33], there are several barriers to and facilitators of implementation. For instance, by interviewing managers, Nilsen et al. [34] found four barriers to implementing palliative care at nursing homes: the staff’s beliefs in their capability to face dying among older persons, the staff’s attitudes to changes at the nursing home and the resources and time required. The barrier regarding the capacity to face dying among older persons has also been shown among nurses’ aides at nursing homes [32].

The target populations for the intervention (factor 4) were the staff and managers at the nursing homes, which meant that next of kin and the older persons were the secondary groups who were expected to receive benefits of the intervention. Our assumption in choosing QoL among next of kin as one outcome measurement was that by implementing palliative care in nursing homes, the next of kin would be affected by receiving support from staff in accordance with a palliative care approach. However, the intervention was given to one group (approximately 8–12 persons) at each nursing home. Depending on the number of staffs at each nursing home (ranging from 23 to 90), the percentage of the staff who received the intervention was between 13 and 50%. This means not only that the results probably differed between the nursing homes but also that the exposure was very low. A potential modification might be to intensify the magnitude of the interventions, for instance, by letting all the staff at the nursing homes receive the intervention. In our study, five meetings over a period of 6 months were implemented. The intervention was designed so that it could be shared with the other staff in the form of tasks to implement between each seminar, but this approach might not have been enough. Other interventions have used the so-called “booster sessions”, i.e. additional sessions conducted periodically to reinforce the knowledge given in the seminars to enhance the intervention effect [35, 36], which is another possible modification of the intervention.

Finally, the context of the implementation (factor 5) refers to the characteristics of the context, i.e. the social environment in which the implementation takes place. In this study, we aimed to investigate the characteristics of the included nursing homes that differed greatly. However, the social environment that impacts the implementation process is often beyond the control of the implementers.

Moreover, there were methodological challenges in this study that will be discussed. These challenges included attrition and the use of outcome measures that were insensitive to change. In this study, the primary attrition occurred at two points: when those who were asked about participation by the contact person at each nursing home and when those who showed interest in participating but said no when the researcher called to obtain informed consent and an address to mail the questionnaires. The secondary attrition, i.e. treatment-correlated attrition, between baseline and follow-up, was 63%. These dropouts (*n* = 113) were related to the older persons’ death, poor health, misunderstandings of whose quality of life was being assessed, unknown reasons or missing data. Although fewer dropouts are generally desirable, it is important to ask whether those who participated in the study were representative of the population of interest; we involved participants from large and small nursing homes in both urban and rural areas, which is a strength of the study.

We used the WHOQOL-BREF instrument [23] to access the next of kin’s self-reported QoL. The instrument is recommended for use in a variety of cultural settings, however, as far as we know, it has never been tested specifically with next of kin of older persons in the context of a nursing home. In a recent psychometric study using baseline data from the KUPA project (Rosén et al. unpublished), the WHOQOL-BREF was found to have acceptable psychometric properties when used to access QoL among next of kin of older persons at nursing homes. However, to our knowledge, the instrument has never been tested to assess responsiveness to change, which is a major methodological weakness in relation to the present study. In the same article, a ceiling effect was suggested to restrict the WHOQOL-BREF’s ability to detect true positive changes in QOL over time (Rosén et al. unpublished). This could have had an impact on the ability to measure increased QoL in our sample.

Some of the next of kin in our study were uncomfortable and surprised that we were interested in their QoL because the focus for them seemed to be the older person and their QoL. This fact revealed that next of kin do not consider themselves as targets of intervention, which might have been different if they were more involved in the intervention. We learned this as we had taken additional quality control steps by including an open question to validate the answers and read the comments of the next of kin.

To verify that the intervention was delivered as designed, and as a part of the evaluation of the KUPA project, the research team plans to conduct a fidelity study using extensive data collected. Examples of data collected are the staff and managers’ verbal and written evaluations after each of the seminars, with the possibility to describe what was good and what needs to be improved. Furthermore, six focus group interviews, about readiness to change their work, with 40 staff and 20 managers, as well as individual interviews with 22 managers were conducted before and after the implementation of the intervention. The results of the fidelity study will show the extent to which the intervention has been implemented as intended.

## Conclusions

Since very few published evaluations have presented the effect of implementing palliative care in a nursing home setting, this study contributes valuable knowledge for the proper planning of an effective implementation model for palliative care. This study evaluated the QoL of next of kin to older persons in nursing homes after implementation of an educational palliative care intervention. The results indicated small statistical changes regarding next of kin’s QoL in favour of the intervention. The lessons learned from the study are to include next of kin in the intervention, in particular, as participants at meetings about next of kin. Another suggestion is to include more meetings about the theme next of kin. Both approaches would bring a stronger focus on the family-centred care into the education component, which this study indicates the need for.
